# Transgenic Increase in N-3/N-6 Fatty Acid Ratio Reduces Maternal Obesity-Associated Inflammation and Limits Adverse Developmental Programming in Mice

**DOI:** 10.1371/journal.pone.0067791

**Published:** 2013-06-25

**Authors:** Margaret J. R. Heerwagen, Michael S. Stewart, Becky A. de la Houssaye, Rachel C. Janssen, Jacob E. Friedman

**Affiliations:** 1 Division of Neonatology, Department of Pediatrics, University of Colorado School of Medicine, Aurora, Colorado, United States of America; 2 Department of Biochemistry and Molecular Genetics, University of Colorado School of Medicine, Aurora, Colorado, United States of America; Virgen Macarena University Hospital, School of Medicine, Spain

## Abstract

Maternal and pediatric obesity has risen dramatically over recent years, and is a known predictor of adverse long-term metabolic outcomes in offspring. However, which particular aspects of obese pregnancy promote such outcomes is less clear. While maternal obesity increases both maternal and placental inflammation, it is still unknown whether this is a dominant mechanism in fetal metabolic programming. In this study, we utilized the Fat-1 transgenic mouse to test whether increasing the maternal n-3/n-6 tissue fatty acid ratio could reduce the consequences of maternal obesity-associated inflammation and thereby mitigate downstream developmental programming. Eight-week-old WT or hemizygous Fat-1 C57BL/6J female mice were placed on a high-fat diet (HFD) or control diet (CD) for 8 weeks prior to mating with WT chow-fed males. Only WT offspring from Fat-1 mothers were analyzed. WT-HFD mothers demonstrated increased markers of infiltrating adipose tissue macrophages (*P<*0.02), and a striking increase in 12 serum pro-inflammatory cytokines (*P*<0.05), while Fat1-HFD mothers remained similar to WT-CD mothers, despite equal weight gain. E18.5 Fetuses from WT-HFD mothers had larger placentas (*P*<0.02), as well as increased placenta and fetal liver TG deposition (*P*<0.01 and *P*<0.02, respectively) and increased placental LPL TG-hydrolase activity (*P*<0.02), which correlated with degree of maternal insulin resistance (*r* = 0.59, *P*<0.02). The placentas and fetal livers from Fat1-HFD mothers were protected from this excess placental growth and fetal-placental lipid deposition. Importantly, maternal protection from excess inflammation corresponded with improved metabolic outcomes in adult WT offspring. While the offspring from WT-HFD mothers weaned onto CD demonstrated increased weight gain (*P*<0.05), body and liver fat (*P*<0.05 and *P*<0.001, respectively), and whole body insulin resistance (*P*<0.05), these were prevented in WT offspring from Fat1-HFD mothers. Our results suggest that reducing excess maternal inflammation may be a promising target for preventing adverse fetal metabolic outcomes in pregnancies complicated by maternal obesity.

## Introduction

The prevalence of pediatric obesity has increased dramatically over the past two decades and is presenting at a progressively younger age, implicating the importance of early life events in children’s long-term metabolic health. Mounting epidemiological evidence suggests that infants born to mothers who are obese and/or consuming hyper-caloric, nutrient-poor diets are particularly at risk for later metabolic disease [1]–[4]. Likewise, numerous studies in animal models demonstrate that maternal obesity and high-fat feeding leads to metabolic impairments in offspring [5]–[14], including obesity, fatty liver disease, insulin resistance and diabetes. Such studies suggest that early fetal and neonatal exposure to the altered metabolic environment of maternal obesity can have long-term negative consequences. However, our knowledge of which specific factors related to maternal obesity and high-fat diet (HFD) are responsible for these consequences, and therefore potential pathways for intervention, remains limited.

Chronic low-grade inflammation is often implicated in the pathogenesis of the metabolic syndrome and insulin resistance in the non-pregnant obese population [15], [16]. In fact, in non-pregnant animal models, dissociating obesity from its characteristic adipose tissue inflammation is a particularly effective means of preventing insulin resistance and other downstream metabolic complications [17]–[22], suggesting that maternal metabolic inflammation may be an important therapeutic target. Previously, in a non-human primate model of maternal obesity, we reported that maternal HFD during pregnancy results in increased fetal hepatic lipid accumulation, oxidative stress, and apoptosis [23], accompanied by a dramatic reduction in fetal plasma omega-3 (n-3) polyunsaturated fatty acids (PUFA) and a more pro-inflammatory cytokine profile [24]. While maternal systemic and placental inflammation are now recognized as prominent features of pregnancies complicated by obesity [25]–[31], little is known about the causal role this inflammation may play in altering fetal-placental developmental programming.

One comparatively simple way to reduce metabolic inflammation is by increasing tissue levels of anti-inflammatory n-3 PUFA relative to pro-inflammatory omega-6 (n-6) PUFA [32]–[35]. This method of reducing low-grade inflammation is clearly demonstrated in the Fat-1 transgenic mouse model, which is capable of endogenously converting n-6 PUFA to n-3 PUFA via ubiquitous expression of a *C. elegans*-derived n-3 desaturase under a β-actin promoter [36]–[38]. The Fat-1 mouse has increased tissue levels of both long and short-chain n-3 PUFA [37] and increased levels of n-3 PUFA-derived pro-resolving lipid mediators [39], which results in reduced inflammation and insulin resistance in the context of high-fat feeding [39]–[41], and protection from tissue damage and disease with other pro-inflammatory challenges [42]–[47], all without any change in dietary n-3 or n-6 PUFA intake.

Many studies have examined the relationship between increased maternal n-3 PUFA supplementation or dietary intake and maternal and infant outcomes [48]–[53]; however, none of these studies examined the effect of re-balancing n-3/n-6 PUFA ratios as an interventional approach to counter the negative effects of obese pregnancy and maternal inflammation on the development of fetal metabolic systems. Further, while previous studies examining interventions such as maternal antioxidant supplementation [51], maternal metformin treatment [48], or neonatal leptin administration [52] are promising, increasing the maternal n-3/n-6 ratio is particularly attractive given that maternal PUFA, especially long chain n-3 PUFA such as docosahexanoic acid (DHA), are already essential nutrients for the developing fetus [49]. Herein, we utilize the Fat-1 transgenic mouse on HFD to demonstrate that increasing the maternal n-3/n-6 PUFA ratio can effectively reduce maternal metabolic inflammation, thereby reversing HFD-associated fetal and placental lipid exposure in the absence of a change in maternal obesity. In addition, we show that protecting the mother from obesity-induced chronic inflammation attenuates the development of long-term metabolic consequences of maternal obesity and HFD in WT offspring. On the basis of these results, we discuss how maternal inflammation may play a direct role in the pathways involved in the metabolic programming of the infant.

## Materials and Methods

### Ethics Statement

All animal studies were approved by the University of Colorado Institutional Animal Care and Use Committee and carried out in strict accordance with the guidelines set forth by the Guide for the Care and Use of Laboratory Animal by the National Institutes of Health.

### Animal Breeding and Diet

Transgenic Fat-1 C57BL6/J male mice were provided courtesy of Dr. J.X. Kang [36], and bred to wild type (WT) C57BL6/J females to obtain roughly 50% transgene-positive mixed litters. Eight-week-old female littermates, either WT or hemizygous Fat-1, were then placed on a 45% kcal HFD (Research Diets D12451) or a 10% kcal control diet (CD, Research Diets D12450B, WT only) and fed ad libitum for 8 wks prior to mating with a WT chow-fed male. Successful mating was determined by post-copulatory plug, and defined as E0.5. Males were removed post-coitous, and mothers were maintained on experimental diet throughout pregnancy and lactation stages. Mothers were weighed weekly from the start of experimental diet, then at day 0.5 and 18.5 of gestation. Pups were analyzed at either embryonic day 18.5 (*n = *12–14 mothers per group) or allowed to deliver, weaned onto CD, and analyzed at 20 wks (*n = *10 mothers per group). Fetal genotype and sex were determined by tail-DNA PCR for presence or absence of Fat-1 and SRY genes, respectively (primers, Table S1), and only WT offspring from Fat-1 mothers were analyzed. For adult offspring studies, litters were standardized to 8 pups on day 1 to control for nutrient bias during lactation. As with fetal offspring, only WT adult offspring were analyzed. Mothers and adult offspring were euthanized by isoflurane anesthesia followed by cervical dislocation and exsanguination. Fetal pups were euthanized by decapitation and exsanguination.

### Tissue Collection

At day 18.5 of gestation, mothers were fasted 4 hrs in a clean cage. Maternal fasting blood was obtained from the tail vein, and glucose levels measured using the Accu-Check Aviva monitoring system (Roche Diagnostics, Indianapolis IN). Mothers were then euthanized by isoflurane anesthesia followed by exsanguination by cardiac puncture. Maternal blood and tissues were quickly collected, rinsed in PBS and processed according to respective analyses listed below. Fetal-placental units were then quickly dissected from the uterine horns, fetal membranes were dissected away, and the placenta and fetus were weighed and measured prior to fetal decapitation and drainage of trunk blood. Fetal livers were then dissected and either embedded in OCT or snap frozen and stored at −80°C. Adult offspring were harvested at 20 wks of age. After a 5 hr fast, animals were euthanized by isoflurane anesthesia followed by exsanguination by cardiac puncture. Blood and tissues were collected and processed as described below.

### Serum Analyses

Blood was collected from mothers, fetuses and adult offspring by cardiac puncture post-anesthesia or decapitation (fetal), and allowed to clot for 10 min at room temperature. Due to low blood volumes in fetal samples, WT pup serum was pooled prior to further analysis. Serum was isolated by centrifugation at 5000 *g* for 10 min, aliquoted and snap-frozen and stored at −80°C. Serum insulin and adiponectin levels were analyzed by Enzyme Immuno Assay (EIA, ALPCO Diagnostics) per manufacturer’s instructions. Serum non-esterified fatty acids (NEFA/FFA)**,** triglycerides (TG) and free glycerol levels were quantified by an enzymatic, colormetric assay (Wako Diagnostic, Richmond VA and Sigma-Aldrich, St. Louis, MO, respectively) per manufacturer’s instructions. All measurements were quantified by microplate reader (Molecular Devices, Sunnyvale CA), using standards provided by the manufacturer. Maternal serum pro-inflammatory cytokine levels were determined using the Mouse Inflammation Antibody Array I (RaBiotech Inc., Norcross, GA) per manufacturer’s instructions. For each membrane, 125 µl of serum from four separate mothers was pooled and diluted 1∶2 with blocking buffer, three membranes (*n* = 12 animals) were used per experimental condition, and results were normalized to the relative expression of IgG positive control internal standards.

### Flow Cytometry for Adipose Tissue and Placental Macrophages

Subsequent to euthanasia, the left perigonadal adipose tissue depot was dissected and rinsed in DMEM containing 10% FBS. Both adipose tissue and whole placentas were minced to 1 mm cubes and digested in the same media containing 500 U/ml Type I Collagenase (Sigma-Aldrich) for 1 hr at 37°C with agitation. The suspension was then filtered through chiffon mesh and the stromal-vascular fraction was isolated by centrifugation at 500 *g* for 5 min. The pelleted fraction was resuspended in 1 X PBS containing 5% BSA. For flow analysis, samples and appropriate controls were stained in 100 µl of 1 X PBS/BSA buffer containing 2 µl Mouse SeroBlock FcR (AbD Serotec, Raleigh, NC). Fluorescently-conjugated primary antibodies (phycoerythrin-F4/80, and allophycocyanin-CD11c, eBioscience, San Diego, CA) or the respective isotype control IgGs (eBioscience) were added after 10 min of blocking, and incubated with cells for 30 min at 4°C. After staining, cells were washed and fixed for 15 min is 1% paraformaldehyde. Cell data was collected using the FACSCalibur flow cytometer and CellQuest Pro Software (BD Biosciences, San Jose, CA), and analyzed using FlowJo software (Tree Star Inc., Ashland, OR). Positive cell populations were expressed as percent positive of total cells, and adjusted by isotype controls.

### Placental RNA Extraction and qPCR

Freshly harvested placentas were equilibrated in RNAlater (Qiagen, Valencia, CA) for 24 hrs prior to storage at −20°C. Thawed samples were homogenized in TRIzol Reagent (Life Technologies, Grand Island, NY) and total RNA was isolated according to manufacturer’s instructions. Real-time quantitative PCR was be performed using iQ SYBR Supermix (Bio-Rad, Hercules, CA) or TaqMan Primer-probe sets (Life Technologies) with primers provided by IDT PrimeTime qPCR Primers (Integrated DNA Technologies). Primers were designed to be specific for exon regions of inflammation and lipid transport-related genes: LPL, CD36, FABP-4, IL-1β, IL-6, TNFα, F4/80, iNOS, and Arg-1 (primers, Table S1). Reactions were run in duplicate on an iQ5 Real-Time PCR Detection System (Bio-Rad) along with a no-template control per gene, and normalized to GAPDH and ubiquitin C expression using the comparative threshold cycling method.

### Placental Lipoprotein Lipase Activity Measurements

Placental lipoprotein lipase (LPL) activity was determined using the methods described by Eckel *et. al.* [54]. Briefly, whole freshly harvested placentas (*n* = 4/mother) were minced in cold Krebs-Ringer-phosphate (KRP) buffer (pH 7.4) to 1–2 mm^3^ pieces. LPL was heparin-released by incubating minced tissue in 0.4 ml of KRP buffer containing 15 µg/ml heparin sulfate. A 100 µl aliquot of free enzyme was removed and incubated with 100 µl of a ^14^C-triolein phosphatidylcholine-stabilized substrate for an additional 45 min at 37°C. The reaction was solubilized and extracted by addition of 3.4 ml of Belfrage solution containing chloroform, methanol and heptane, followed by addition of 0.96 ml of a bicarbonate buffer and agitation. The ^14^C-labeled fatty acids were partitioned by centrifugation for 20 min at 4°C. A 500 µl aliquot of the resulting aqueous supernatant containing LPL-released FFAs was counted by β-scintillation (LS6000TA; Beckman Coulter, Brea, CA). ^14^C-oleic acid was used to control for extraction efficiency, and individual results were normalized to a heparinized rat plasma internal standard.

### Placenta and Fetal Liver Lipid Analysis by GC-MS

Frozen placenta and fetal liver samples were lyophilized overnight prior to lipid extraction, and dry weights recorded. Lyophilized samples were then homogenized in 1 ml of ice-cold methanol, and lipids were extracted using 1∶2 methanol:chloroform followed by 0.6 volumes of distilled water, and solubilization for 1 hr end-over-end at 4°C. The polar and non-polar phases were then separated by centrifugation, and the lower non-polar phase was isolated and dried under nitrogen. Resuspended samples were separated by solid phase extraction (SPE) using a Supelco aminopropyl phase column (Sigma-Aldrich, St. Louis, MO). Phospholipid and TG fractions were methylated using 0.5 M sodium methoxide in methanol, and fatty acid methyl esters (FAMEs) were analyzed by gas chromatography-mass spectrometry (GC-MS) using a DB-23 column and helium as a carrier gas. Temperature was programmed from 140−240°C at 15°/min. Results were normalized to internal extraction standards and resulting concentrations were normalized to dry tissue weight.

### Adult Offspring Liver Triglyceride Quantitation

Flash-frozen adult offspring livers samples were homogenized in 1 ml of ice-cold methanol, and lipids were extracted using 1∶2 methanol:chloroform followed by 0.6 volumes of distilled water, and solubilization for 1 hr end-over-end at 4°C. The polar and non-polar phases were then separated by centrifugation, and the lower non-polar phase was isolated and dried under nitrogen. The dried samples were then resuspended in isopropanol +2% Triton X-100 for direct TG quantitation using Infinity Triglycerides Reagent (Thermo Scientific, Waltham, MA) and a standard curve using glycerol standard solution (Sigma-Aldrich) per manufacturer’s instructions. Resultant TG concentrations were normalized to starting tissue weight.

### Tissue Histology

To visualize lipid droplet deposition, placentas (in cross-section) and fetal and adult offspring livers were cryoembedded in Tissue-Tek OCT Compound (Sakura Finetek U.S.A., Inc., Torrence, CA). 5 µM cryosections were stained using a 1% Oil Red O in propylene glycol solution. Slides were fixed in ice-cold formalin, equilibrated in 100% propylene glycol, and stained for 10 min in pre-warmed 1% Oil Red O solution. Stained sections were then differentiated in 80% propylene glycol, rinsed in distilled water, and counterstained with Mayer’s Hematoxylin (Sigma-Aldrich).

### Adult Offspring Metabolic Analyses

Offspring weight gain was recorded at weaning and every-other week thereafter. At 20 weeks of age animals were fasted 5 hrs, fasting blood glucose was obtained from the tail vein and recorded by glucometer, and an insulin tolerance test (ITT) was performed. Briefly, a bolus of recombinant insulin was injected IP at 0.0075 U/kg body weight, and blood glucose readings were taken from the tail at 0, 10, 20, 30, 45, 60, 75, and 90 mins post-injection. Body composition (fat and lean mass) was determined 3 days post-ITT by EchoMRI™ Body Composition Analyzer (EchoMRI, Houston, TX).

### Data Analysis

To correct for a common maternal environment, results for both fetal and adult offspring were expressed as an average for each mother, with siblings treated as replicates. Only WT placentas, fetuses, and adult offspring from Fat-1 mothers were included in these averages. For fetal phenotyping, male and female data were analyzed separately, and pooled after no between-group sexual dimorphism was identified, while analyses in adult offspring were separated by sex. Differences between maternal groups (and their offspring) were determined by 1-way ANOVA with Bartlett’s test for equal variance using the Graph Pad Prism 5.0 software (GraphPad Software Inc., La Jolla, CA). When variances were not equal, a non-parametric Kruskall-Wallis test and Gausian approximation were used. Post-hoc Student’s *t* tests were performed when necessary to identify between group differences. For experiments involving a time course (weight gain, ITT), a 2-way ANOVA with time as a repeated measure variable was used, with Bonferonni post-tests to compare replicate means. All data are expressed as the geometric mean ± standard error of mean (SEM), and differences were determined to be significant at *P*<0.05.

## Results

### Maternal Obesity-associated Inflammation is Suppressed by Fat-1 Transgene

**Figure 1:**
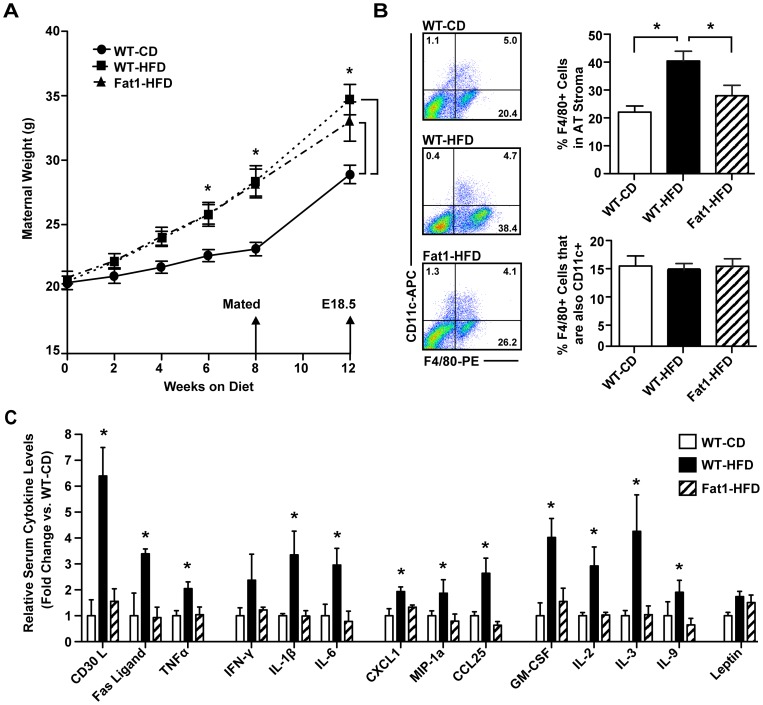
Impact of maternal HFD and Fat-1 transgene on maternal weight gain and inflammatory markers. (A) Maternal weight gain prior to mating, and at E18.5 (late gestation). Gestational weight was estimated by subtracting the complete fetal-placental litter weight from the weight of the pregnant mother. (B) Adipose tissue (AT) macrophage quantitation by flow cytometry. Antibodies against mouse macrophage marker F4/80 and M1 macrophage marker CD11c were used to determine the percent of macrophages present and their relative M1 polarization in maternal AT stroma at E18.5. (C) Relative maternal serum pro-inflammatory cytokine levels at E18.5 by membrane-bound antibody array. Results are the average of three membranes per maternal group, with each membrane incubated with the pooled serum from four separate mothers. (A–C) Results are the average of *n* = 12−14 mothers per experimental group. Data represented as mean ± SEM; *P<0.05.

WT and Fat-1 female littermates were placed HFD for 8 weeks prior to mating to establish an obesity phenotype. A third group of WT female mice were fed a low-fat CD to serve as baseline controls. After 8 weeks on diet, females were mated to WT chow-fed males, and separated to individual cages during gestation. Weight gain was significantly increased in HFD mothers, and importantly, Fat-1 and WT mice gained similar weight on the HFD prior to mating and during pregnancy relative to CD mothers (*P*<0.05; Figure 1A). To determine the effects of *fat-1* expression on inflammation during pregnancy, we assessed both adipose tissue macrophage content and serum pro-inflammatory cytokine levels in late gestation (E18.5) mothers. Despite similar weight gain on HFD, only WT-HFD mothers demonstrated increased adipose tissue macrophage content, as measured by percent F4/80+ cell population in stromal fractions by flow cytometry (*P*<0.02; Figure 1B). However, no difference in M1 polarization of macrophages was observed between groups based on CD11c co-positivity, a previously established M1 phenotypic marker [55]. To determine the effect of HFD on circulating pro-inflammatory cytokine levels, we utilized the Mouse Inflammation Antibody Array. WT-HFD mothers showed strikingly higher levels of 12 circulating pro-inflammatory cytokines (*P*<0.05, Figure 1C), while Fat1-HFD mothers were protected from this pro-inflammatory response to HFD. Maternal leptin levels, while trending higher in HFD mothers, were not significantly elevated.

**Figure 2:**
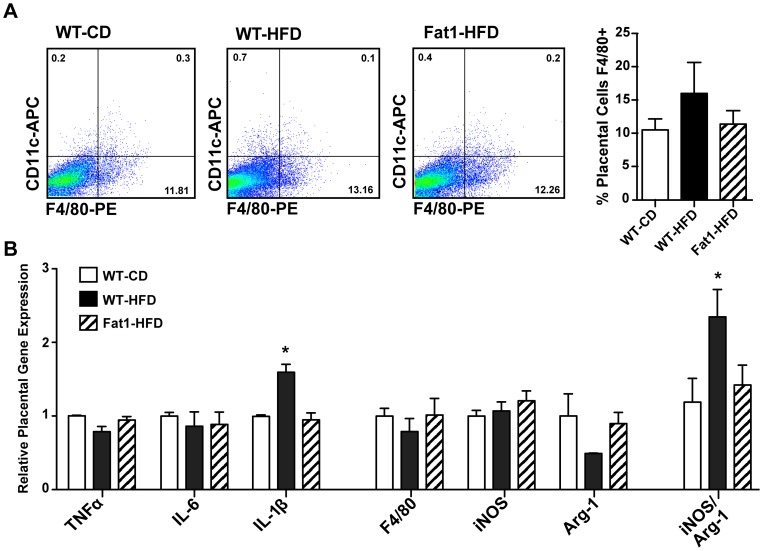
Effects of maternal HFD and Fat-1 transgene on measures of placental inflammation. (A) Flow cytometry for F4/80+ and CD11c+ macrophages in collagenase-digested whole placentas (*n = *6 mothers per group, average of 2 placentas per mother). (B) Placental gene expression of select pro-inflammatory cytokines and macrophage markers by quantitative PCR (*n* = 9 mothers per group, 3 RNA pools of 3 mothers each). Data represented as mean ± SEM with only WT placentas included in maternal averages for the Fat1-HFD group; *P<0.05.

We were not able to identify any increases in macrophage content in the whole placentas of HFD mothers by flow cytometry for F4/80+ cells (Figure 2A), or by F4/80 gene expression (Figure 2B). However IL-1β expression and the ratio of arginase-1 (Arg-1) to iNos expression were significantly increased in WT-HFD placentas (*P*<0.05; Figure 2B), suggesting a potentially more pro-inflammatory microenvironment on HFD, which was again normalized in placentas from Fat1-HFD mothers.

The increase in maternal inflammation in WT-HFD mothers also corresponded with an elevation in fasting insulin levels, and a consequent increase in HOMA-IR score (*P*<0.05 and *P*<0.01, respectively; Table 1). Fat1-HFD mothers’ insulin levels and HOMA-IR score did not differ significantly from WT-CD mothers. While maternal fasting glucose levels in WT-HFD mothers trended higher, no groups demonstrated overt fasting hyperglycemia (Table 1). Maternal fasting TG levels did not differ between groups (Table 1), but maternal fasting free fatty acid (FFA) levels were lowered in WT-HFD mothers versus both WT-CD and Fat1-HFD mothers (*P*<0.02; Table 1). No differences in serum high molecular weight (HMW) adiponectin were observed between maternal groups.

**Table 1:** Maternal E18.5 fasting serum measurements.

Maternal Group	Glucose (mg/dL)	Insulin (ng/mL)	HOMA-IR	FFA (mq/L)	TG (mg/dL)	Glycerol (mg/dL)
**WT-CD**	95±6	0.41±.06	2.12±0.26	0.98±0.05	22.98±3.97	15.51±0.61
**WT-HFD**	117±7	*0.83±0.13	*6.21±1.34	*0.75±0.04	18.23±1.31	15.46±0.87
**Fat1-HFD**	104±6	0.53±0.08	2.86±0.61	0.86±0.06	18.62±2.85	16.46±0.91

Data are expressed as mean ± SEM of *n* = 12–14 mothers per maternal group; *P<0.05 *vs.* WT-CD and Fat1-HFD.

### Maternal HFD Reduces Fetal/Placental Ratio, Ameliorated in Fat-1 Mothers

**Figure 3:**
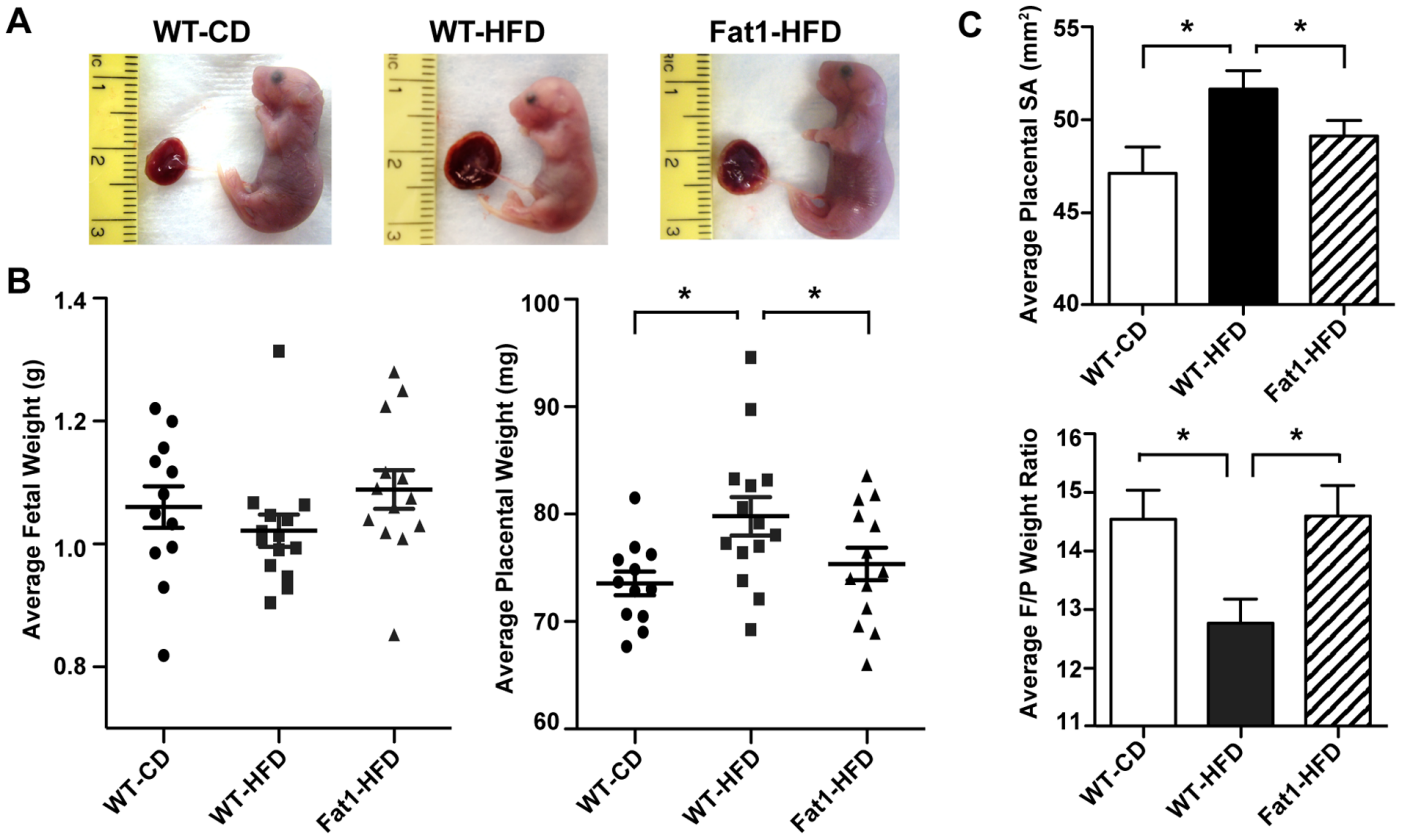
Effect of maternal HFD and Fat-1 transgene on placental and fetal gross measurements. (A) Representative images of fetal-placental unit size for each maternal group. (B) Average fetal and placental weights for each litter. Placentas were weighed subsequent to removal of umbilical cord and fetal membranes. (C) Average calculated placental surface area (SA) and fetal/placental (F/P) weight ratio for each litter. Placental surface area was calculated from measured diameter on decidual side. All data are expressed as mean ± SEM of the litter average for each mother (*n* = 12−14 mothers per experimental group), with only WT fetal-placental units included in maternal averages for the Fat1-HFD group; *P<0.05.

Fetuses from WT-HFD mothers had significantly larger placentas versus WT-CD mothers at E18.5, as demonstrated by both an increase in placental weight and surface area (*P*<0.02; Figure 3A–C). There was no difference in litter male/female sex ratio between groups; however, litter size was reduced from an average of 10 to 8 pups in both HFD groups (data not shown). Fetal weights in WT-HFD mothers trended lower than in WT-CD mothers (Figure 3B), leading to a reduced fetal-placental ratio overall (*P*<0.01; Figure 3C). This pattern suggests reduced placental efficiency and restricted fetal growth relative to placental size, and was reversed in fetuses from Fat1-HFD mothers. While fetal sex significantly affected placental and fetal weights in all maternal groups, no sexual dimorphism was apparent with respect to maternal HFD (data not shown). Despite differences in growth in WT-HFD fetuses, there were no significant changes in pooled fetal serum levels of glucose, insulin, TG, or FFA between maternal groups (Table 2). Given the low volume of fetal serum, we attempted to use a multiplex ELISA to detect fetal levels of the pro-inflammatory cytokines IL-1β, IL-6, and TNFα, but levels in all experimental groups at E18.5 were below the detectable range of the assay and therefore are not reported here.

**Table 2:** Fetal E18.5 fasting serum measurements.

Maternal Group	Glucose (mg/dL)	Insulin (ng/mL)	FFA (mq/L)	TG (mg/dL)	Glycerol (mg/dL)
WT-CD	116±16	1.78±0.47	0.11±0.02	26.53±1.78	6.58±0.72
WT-HFD	95±9	1.35±0.17	0.09±0.01	26.90±1.00	5.45±0.52
Fat1-HFD	102±12	1.88±0.44	0.11±0.01	24.89±2.85	4.56±0.64

Measurements were conducted on pooled litter serum for each mother. Data are expressed as mean ± SEM for the fetal serum of *n* = 12−14 mothers per maternal group.

### Maternal *fat-1* Expression Protects Against HFD-associated Placental and Fetal Lipid Accumulation

**Figure 4:**
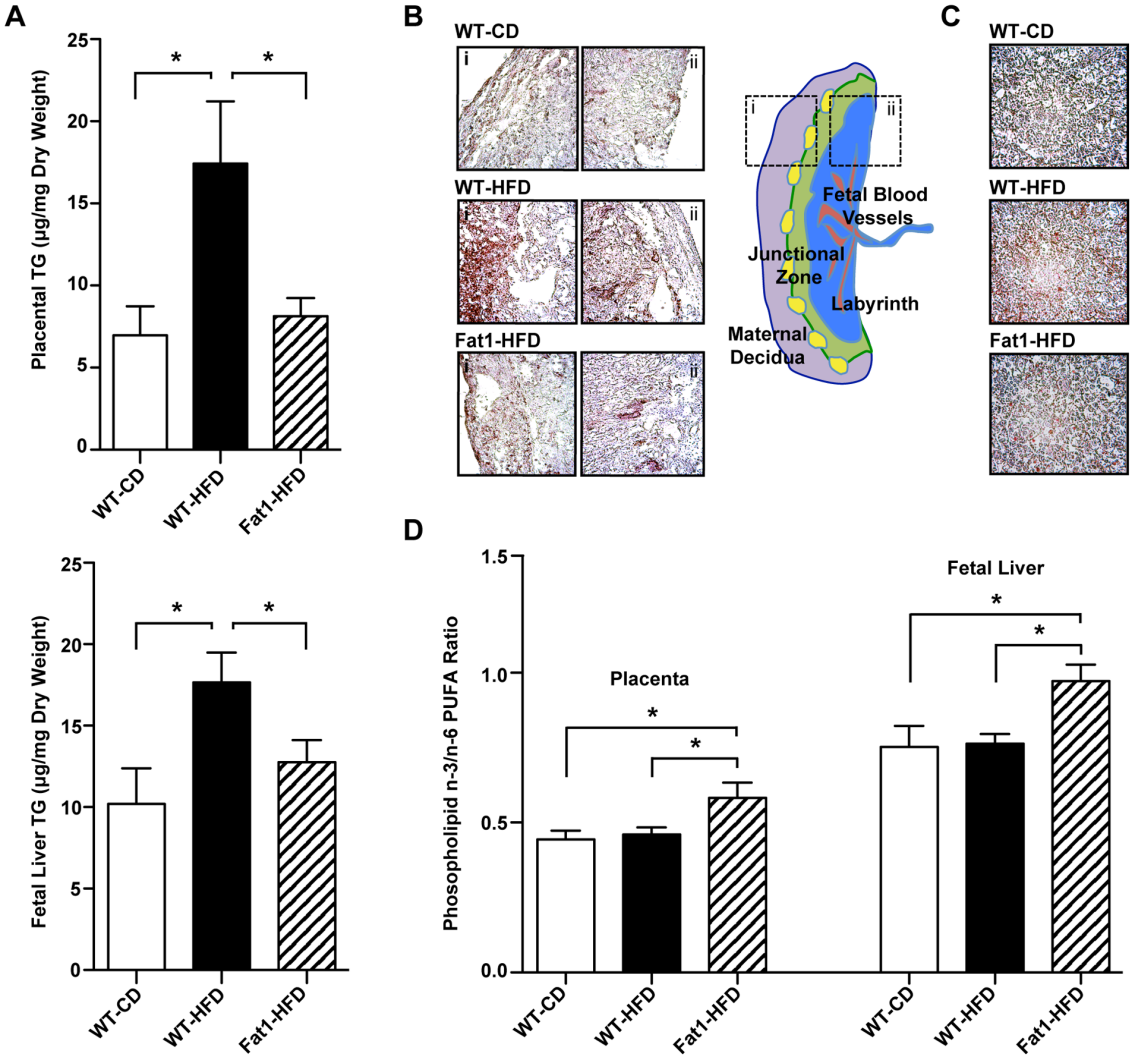
Effects of maternal HFD and Fat-1 transgene on placental and fetal liver TG deposition and phospholipid n-3/n-6 ratio. (A) Quantification of placental and fetal liver TG by GC-MS on extracted tissue TG lipid fractions (*n = *6 maternally unique placenta-fetal liver pairs per experimental group). (B and C) Oil Red O staining of representative placenta (B) and fetal liver (c) cryosections to localize TG deposition quantitated in (A) (lipid droplet = red, cell nuclei = blue, 10X magnification). For placentas, representative cross-sectional images were captured of decidual (i) and labyrinth (ii) sides, as indicated in drawing. (D) Placental and fetal liver n-3 and n-6 PUFA ratio, as determined by GC-MS characterization of tissue phospholipid fractions to measure relative levels of n-3 and n-6 PUFA. All data are expressed as mean ± SEM of the litter average for each mother (*n = *6 mothers per experimental group), with only WT fetal-placental units included in maternal averages for the Fat1-HFD group; *P<0.05.

To better identify cumulative differences in fetal-placental lipid exposure over gestation, GC-MS was performed on placenta and fetal liver lipid fractions. Both the placenta and fetal livers from WT-HFD mothers showed increased TG levels relative to WT-CD and Fat1-HFD mothers (*P*<0.01 and *P*<0.02, respectively; Figure 4A). This increased TG was confirmed by Oil Red O staining of tissues in cross-section, with placental lipid accumulation primarily in the decidual zone (Figure 4B), and diffuse accumulation throughout the fetal liver (Figure 4C). In addition, tissue differences in n-3/n-6 PUFA ratios were examined in placenta and fetal liver phospholipid fractions, where the majority of PUFA localize. While maternal HFD did not impair n-3 PUFA transfer, as seen by an equal stepwise increase in n-3/n-6 ratios from the placenta to the fetus in WT-CD and HFD mothers (Figure 4D), maternal *fat-1* transgene expression did increase both placental and fetal liver n-3/n-6 ratios, primarily due to greater levels of the long chain n-3 PUFA DHA (Figure 4D).

**Figure 5:**
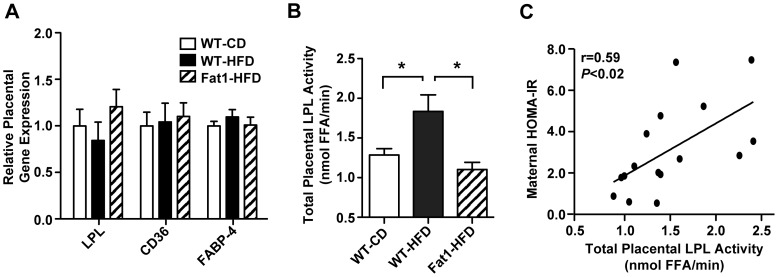
Effect of maternal HFD and Fat-1 transgene on placental fatty acid transporter expression and lipoprotein lipase activity. (A) Placental gene expression of select genes involved in fatty acid transport by quantitative PCR (*n = *9 mothers per group, 3 RNA pools of 3 mothers each). (B) Placental LPL hydrolase activity. Activity was measured in 3–4 placentas from each mother and averaged, with *n* = 6 mothers per experimental group. (C) Correlation of average maternal placental LPL activity and maternal insulin resistance, as measured by HOMA-IR. All data are expressed as mean ± SEM with only WT placentas included in maternal averages for the Fat1-HFD group; *P<0.05.

In order to clarify whether this difference in lipid accumulation could be due in part to differential lipid uptake we examined the expression of a limited set of genes related to lipid transport, but no changes in placental LPL, CD36, or FABP-4 expression were observed (Figure 5A). Nevertheless, given that the regulation of placental transport is often post-transcriptional and affected by environmental changes, placental LPL activity was assayed on freshly dissected E18.5 placentas from a subset of mothers. In line with the increased placenta and fetal liver TG deposition observed in WT-HFD mothers, placental LPL activity was significantly elevated in WT-HFD mothers (*P*<0.02; Figure 5B), with Fat1-HFD mothers again comparable to WT-CD. Importantly, this elevated placental LPL activity observed significantly correlated with maternal HOMA-IR score among all groups (*r* = 0.59, *P*<0.02; Figure 5C).

### WT Offspring from Fat-1 Mothers Selectively Protected from Adverse Metabolic Programming

**Figure 6:**
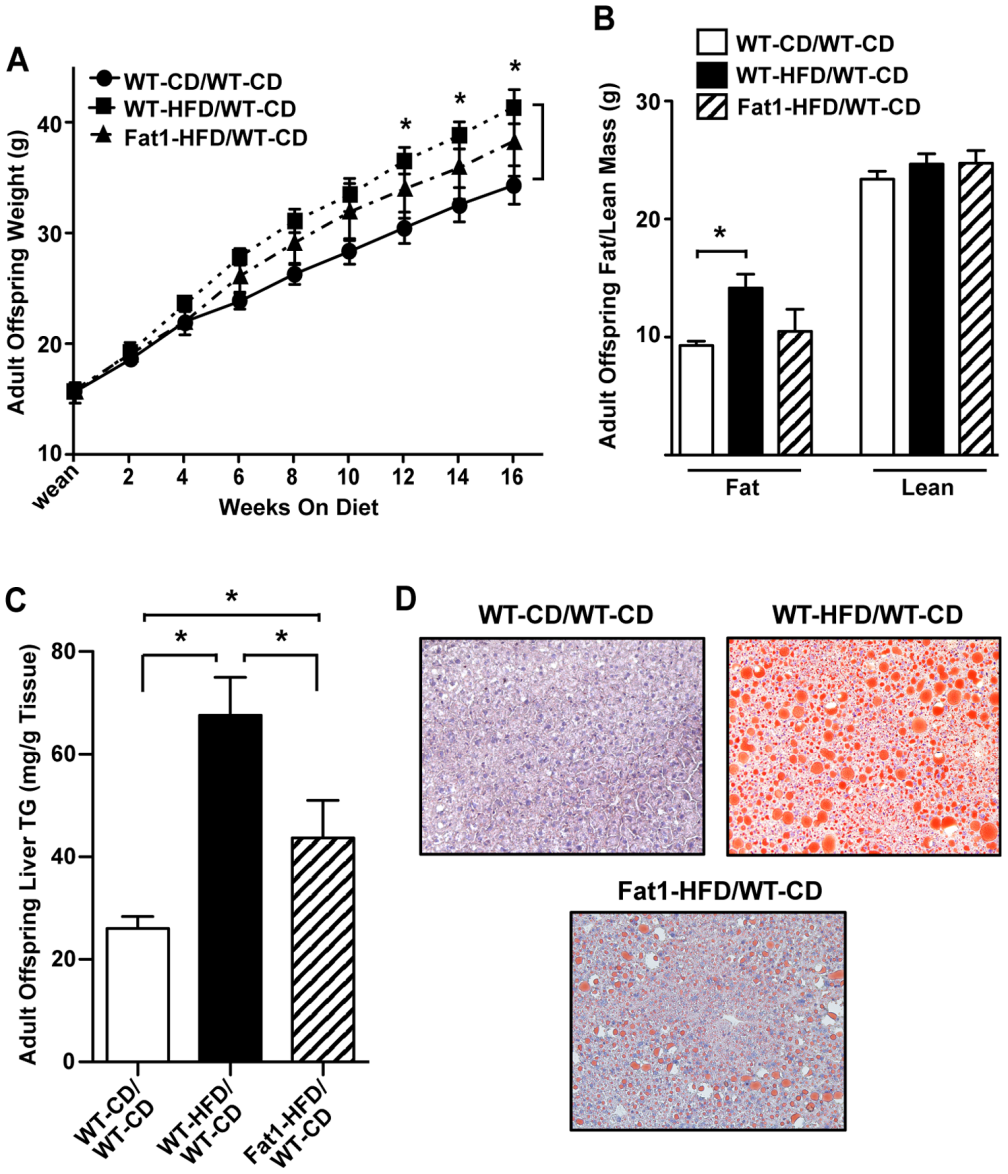
Effect of maternal HFD and Fat-1 transgene on WT adult male offspring weight gain and body composition. (A) WT male offspring weight gain. Offspring from each maternal group (*n* = 10 mothers per group) were weaned onto CD, and weighed every other week for 16 weeks. (B) Adult male offspring body composition (fat and lean mass) at 16 wks post-wean, as determined by Echo MRI. (C and D) Adult male offspring liver TG deposition. TG were quantitated in (C) by colorimetric assay on tissue lipid extracts, and visualized in (D) by Oil Red O staining of representative liver cryosections for neutral lipids (lipid droplet = red, cell nuclei = blue, 20X magnification). All offspring measures are averaged per mother, with siblings treated as replicates. For Fat-1 mothers, only WT offspring were included in analyses. All data are expressed as mean ± SEM; *P<0.05.

To determine whether the protective properties of maternal *fat-1* expression on HFD extended into adult offspring, WT pups from all mothers were weaned onto low-fat CD (“maternal genotype – maternal diet/WT-CD”) and analyzed at 20 wks of age. Both male and female offspring demonstrated similar patterns in terms of programmed weight gain and fat mass. However, the difference in weight gain between female WT-CD/WT-CD and WT-HFD/WT-CD offspring was not as robust in comparison to males, with a non-significant trend for increased fat mass, and maintenance of insulin sensitivity in all groups (Figure S1). Because of this, only male offspring were further examined for associated pathology. In both sexes, offspring weight did not differ between maternal groups at weaning; however, by 16 wks post-weaning male and female WT-HFD/WT-CD offspring were significantly heavier relative to WT-CD/WT-CD offspring (*P*<0.05; Figure 6A). While Fat1-HFD/WT-CD offspring demonstrated a pattern of increased weight gain relative WT-CD/WT-CD offspring, this difference was not significant. Further, male WT-HFD/WT-CD offspring had increased fat mass (*P*<0.05; Figure 6B) and liver TG deposition (*P*<0.001, Figure 6, C and D) at 20 wks of age, while male WT-CD/WT-CD and Fat1-HFD/WT-CD offspring demonstrated similar body compositions and significantly reduced liver TG relative to WT-HFD/WT-CD offspring. Lean mass did not differ between groups (Figure 6B, Figure S1).

**Figure 7:**
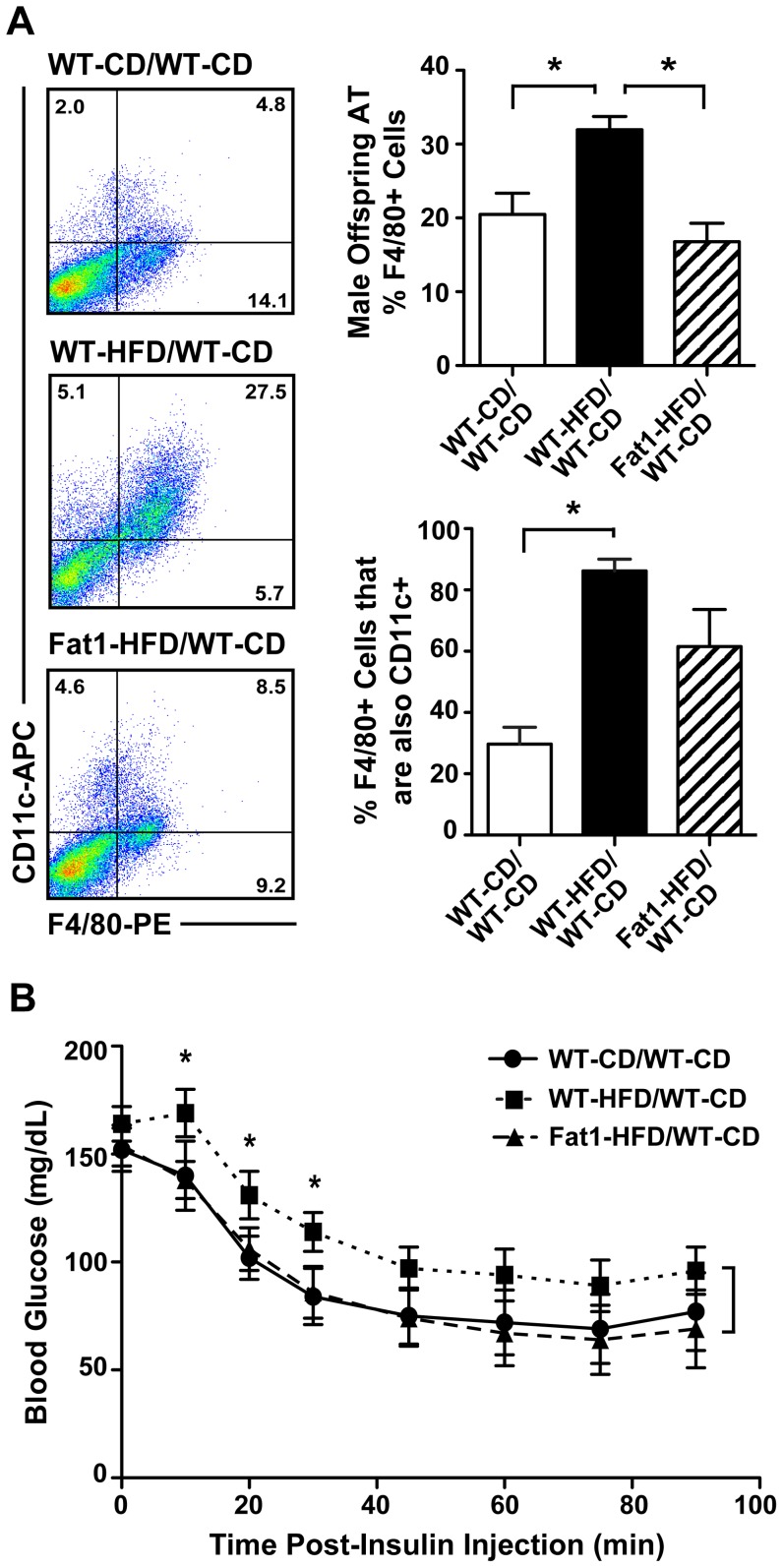
Effect of maternal HFD and Fat-1 transgene on WT adult male offspring metabolic inflammation and insulin resistance. (A) Adipose tissue (AT) macrophage quantitation by flow cytometry. Antibodies against mouse macrophage marker F4/80 and M1 macrophage marker CD11c were used to determine the percent of macrophages present and their relative M1 polarization in adult offspring AT at 16 wks post-weaning. (B) Blood glucose curve in response to insulin tolerance test on fasted adult male offspring 16 wks post-weaning. All offspring measures are averaged per mother, with siblings treated as replicates. For Fat-1 mothers, only WT offspring were included in analyses. All data are expressed as mean ± SEM; *P<0.05.

Given the greater weight gain, adiposity and liver TG deposition in male WT-HFD/WT-CD offspring, obesity-associated inflammation and insulin resistance were also assessed. Adipose tissue depots of WT-HFD/WT-CD male offspring showed greater macrophage content, as well as increased M1 polarization (*P*<0.001 and *P*<0.01, respectively; Figure 7A) relative to WT-CD/WT-CD offspring. This pattern of increased adipose tissue inflammation was not observed in Fat1-HFD/WT-CD offspring. Differences in insulin sensitivity were determined by insulin tolerance test (ITT). Male WT-HFD/WT-CD demonstrated a reduced insulin response relative to WT-CD/WT-CD and Fat1-HFD/WT-CD offspring (*P*<0.05; Figure 7B), who displayed markedly similar serum glucose curves. Additionally, while fasting serum glucose levels did not differ between groups, serum insulin levels were elevated in male WT-HFD/WT-CD offspring only (*P*<0.02; Table 3), further supporting a programmed increase in insulin resistance in male WT-HFD/WT-CD offspring, with Fat1-HFD/WT-CD offspring showing levels more similar to WT-CD/WT-CD offspring.

**Table 3:** Adult WT offspring fasting serum measurements.

Adult Offspring Group	Glucose (mg/dL)	Insulin (ng/mL)	FFA (mq/L)	TG (mg/dL)	Glycerol (mg/dL)
WT-CD/WT-CD	151±10	2.04±0.52	0.76±0.13	92.4±9.7	49.1±3.6
WT-HFD/WT-CD	164±8	*6.44±1.71	0.59±0.06	116.3±6.5	47.1±3.9
Fat1-HFD/WT-CD	154±9	3.53±0.98	0.51±0.06	111.1±9.0	41.0±2.3

Measurements were performed on the 20 wk old fasting serum collected from male adult offspring sampled from *n = *8 mothers per experimental group. All offspring were weaned onto CD. Data are expressed as mean ± SEM; *p<0.05 *vs.* WT-CD and Fat1-HFD.

## Discussion

Maternal pre-gravid obesity increases the risk for numerous obstetric complications such as preeclampsia and gestational diabetes mellitus [56], [57], but also increases sub-clinical risk factors, such as maternal and placental inflammation [25]–[30]. While inflammation and insulin resistance are well-recognized characteristics of both pregnancy and obesity, little is known about the mechanisms linking these maternal changes with fetal-placental health or, perhaps more importantly, the pathogenesis of obesity in offspring. Our previous research in non-human primates suggests that both maternal obesity and high-fat feeding increase fetal exposure to lipids and pro-inflammatory cytokines [23], [24], which we hypothesize may originate from maternal and placental inflammation and inflammation-associated insulin resistance. In the current study, we used the Fat-1 mouse model to demonstrate that a targeted intervention to reduce obesity-associated inflammation by increasing the maternal n-3/n-6 tissue fatty acid ratio can prevent such an exposure without any manipulation of maternal weight, thereby reducing metabolic impairments in both the fetus and future adult offspring.

Our findings demonstrate that maternal expression of the *fat-1* transgene protects mothers from increases in obesity-associated F4/80+ macrophage accumulation in adipose tissue depots, and systemic increases in pro-inflammatory cytokines associated with immune cell activation, proliferation and chemotaxis. Importantly, this protection occurred despite equal weight gain between WT and Fat-1 HFD-fed mothers. Maternal inflammation was also associated with increased maternal insulin resistance, as indicated by increased maternal fasting insulin levels and estimated by the homeostasis model of assessing insulin resistance (HOMA-IR). These findings of increased low-grade inflammation and reduced insulin sensitivity without overt hyperglycemia are consistent with the progression of obesity-related metabolic syndrome in non-pregnant mouse models [15], [16], [26], and the protection afforded to these models by the *fat-1* transgene [39]–[41]. Surprisingly, we did not detect any increase in F4/80+ macrophage accumulation in the placenta of HFD mothers, as has been previously reported in human pregnancies complicated by obesity [30]. However, our methods involved utilization of the whole placenta, and therefore may simply have lacked the sensitivity to detect regional changes in cell populations. Increases in IL-1β and reduced Arg-1 relative to iNos gene expression in WT-HFD mothers still suggested a more inflamed placental phenotype overall, and were consistent with previous findings in primates and humans [26], [27], [30]. Importantly, Fat1-HFD mothers did not show this same placental pro-inflammatory pattern.

In accordance with previous groups, our mouse model of obese pregnancy produced fetuses with mild evidence of growth restriction [23], [58], [59]. While these findings are somewhat contrary to the large for gestational age (LGA) infants common to human pregnancies complicated by obesity, this may simply be due to species-specific differences in developmental age and fat mass at birth. However, recent studies in human pregnancy suggest that infant body composition, specifically increased adiposity and intrahepatic fat accumulation [12], [60], [61], may be more relevant than birth weight to overall infant health. More concerning than the trend in reduced fetal growth was the evidence of reduced placental efficiency, with WT-HFD mothers producing slightly smaller fetuses with significantly larger placentas [62]. This reduced fetal-placental ratio was recently reported by another group [63], and may be indicative of previously described impairments in placental perfusion and vascular defects related to maternal obesity and HFD [26], [27]. Contrary to our findings, some groups have observed reduced placental size with maternal HFD [58], which may be partially explained by differences in mouse strains, diet composition, duration of feeding, gestational age at time of harvest, as well as maternal age at time of conception [64]. Clearly, the maternal environment plays a key role in the proper establishment, growth, and function of the placenta [65], and the mechanisms by which maternal obesity and HFD can impair these processes requires further examination.

Corresponding with our findings in the fetal liver of non-human primates, and with previous groups’ findings in the human and rodent placenta [66], [67], maternal HFD led to a significant increase in lipid deposition in the decidua, and to a lesser extent, the labyrinth regions of the placenta, as well as in the fetal liver, which was mitigated in Fat1-HFD mothers. This was observed despite no evidence of late gestation maternal hyperlipidemia based on fasting serum measurements. It is well established that decidua cells have the capacity to store lipid, as this is one of the characteristic features of the decidual reaction in early gestation, while cells within the murine placental labyrinth are primarily involved in nutrient exchange rather than storage, which may explain the differential TG deposition between placental regions. Importantly, we did not observe any evidence of impaired n-3 PUFA transport, as our earlier findings in fetal plasma from non-human primates suggested [24]. However, we were only able to assess fetal tissue levels, due to the very low volume of fetal mouse plasma. Fetuses from all maternal groups had relatively high n-3 PUFA phospholipid levels compared to maternal diet, consistent with the theory that the placenta has a preferential uptake mechanism for n-3 PUFA specifically and maternal PUFA in general relative to other lipid species [68]–[70]. It is important to note that we detected a significant increase in placental and fetal liver phospholipid DHA levels, and thus an increased n-3/n-6 PUFA ratio in WT offspring from Fat-1 mothers, which cannot be ruled out as a second potential mechanism of protection against adverse programming.

Previous groups have reported an association between maternal obesity and increased placental expression of LPL and various other fatty acid transporters and binding proteins [66], [67], [71]–[74]. However, we detected no differences in LPL, nor CD36 or FABP-4, at the level of expression, but instead WT-HFD mothers demonstrated increased LPL enzymatic activity, which correlated with degree of maternal insulin resistance. Given that LPL is both an established gatekeeper in the tissue uptake of lipoprotein particles, and known to be regulated at a post-translational and tissue-dependent level [75], [76], it follows that alterations in the maternal endocrine environment, such as increased inflammation and insulin levels, could impact its hydrolase activity [65], [76].

Critical to the above findings was the question of whether the protective effects observed in Fat1-HFD mothers had a long-term impact on offspring metabolic health, so we assessed many previously reported programming outcomes in the adult offspring from a second cohort of mothers [5]–[10]. Male WT offspring from Fat1-HFD mothers demonstrated less weight gain, adiposity, fatty liver, adipose tissue macrophages, hyperinsulinemia, and insulin resistance. In the majority of measures, Fat1-HFD/WT-CD offspring did not significantly differ from controls; however some trends in programming were not completely reversed, but rather mitigated. This underscores the complexity of the maternal-fetal obesity paradigm; where it is likely that many factors related to maternal obesity and HFD, in addition to the metabolic inflammation reported here, also impact the development of fetal metabolic systems. Importantly, despite similar trends, we did not observe the same degree of severity in programming in female offspring as we did in males. Whether this is due to actual differences in programming, or instead baseline sex differences in tendency towards metabolic disease, still remains to be clarified.

Our findings illustrate the potential to reduce adverse fetal programming events by indirectly limiting maternal inflammation to improve the metabolic environment in which the placenta and fetus develops. Transgenic increase of maternal n-3/n-6 PUFA reduces obesity-related inflammation, which is associated with improved maternal insulin sensitivity and reduced placental and fetal hepatic lipid accumulation. The mechanism by which excess cytokines and lipids can program fetal metabolic systems is still being elucidated, and there are likely many tissue targets and mechanisms. While the increased placenta and fetal hepatic lipid droplets we observed are a method of cellular lipid storage and are not inherently damaging, our previous findings of increased fetal hepatic lipotoxicity and oxidative stress [23] and results on the programming of fatty liver in adult offspring [5], [13], [14], [77], [78] suggest that an aberrant influx of lipids into a still-developing tissue can have both short- and long-term consequences on tissue function [79]. This is not to say, however, that the mechanisms of maternal HFD programming are localized to the fetal liver. On the contrary, the effects are likely global, and impact the regulation of many inter-connecting systems, including nutrient metabolism, insulin signaling, and appetite/satiety pathways.

To our knowledge, this is the first time that maternal n-3/n-6 PUFA status and HFD have been studied in combination, with a focus on preventing the adverse metabolic programming attributed to maternal obesity. Utilization of the Fat-1 mouse allows for the isolation of the n-3/n-6 PUFA variable in otherwise genetically identical sibling females, and without any other change in maternal obesity. However, use of a transgene prevents us from ruling out protective effects that may be established during early development and puberty, for instance improved oocyte quality. While we sought to reduce the relative impact of this by exposing mothers to HFD only after they had reached adulthood, early benefits of the *fat-1* transgene cannot be eliminated. Additionally, we cannot delineate whether it is the increase in n-3 PUFA or the reduction in n-6 PUFA that is most important. Both of these essential fatty acids play an important role in fetal development, as indicated by the preferential uptake of long chain PUFA by the placenta [68], [70], [80]. Studies have associated maternal n-6 PUFA status with increased adiposity in childhood [81], and n-3 PUFA status with beneficial effects on fetal growth and placental function [50] and reduced adiposity [82]. However, the overall impact of balancing maternal n-3/n-6 PUFA status is still unclear [83], particularly as none of these studies include obese pregnancy.

It is likely that both the excess of n-6 PUFA and paucity of n-3 PUFA, characteristic of more Western-style high-fat diets, play a functional role in adverse obese pregnancy outcomes. Multiple mechanisms for the anti-inflammatory nature of n-3 PUFA have been suggested, including inhibition of macrophage activation by the binding of G protein-coupled receptor 120 (GPR120) [33], and the potent inflammation-resolving effects of n-3 PUFA derived lipid mediators [84]. Still, one of the most straightforward mechanisms for the effects of n-3 fatty acids is their inherent competition with n-6 fatty acids for incorporation into phospholipid membranes. Given that n-3 and n-6 PUFA are both broken down to their most biologically active eicosanoid products by the same enzymatic pathways, it follows that a 1∶1 ratio of the two is ideal [84], [85]. Thus, potential therapies should focus on not only increasing maternal n-3 PUFA intake, but correspondingly reducing dietary n-6 PUFA and overall saturated fat intake as well.

In summary, our findings demonstrate a novel mechanism to reduce maternal inflammation and insulin resistance in a mouse model of obese pregnancy. Targeting early maternal metabolic inflammation by increasing maternal n-3/n-6 PUFA status reduces fetal-placental lipid exposure, and limits the development of adverse metabolic outcomes in adult offspring. Our results suggest that early intervention at the level of maternal inflammation and dietary fat balance may be a promising target for future preventative therapy in pregnancies complicated by obesity.

## Supporting Information

Figure S1 **Effect of maternal HFD and Fat-1 transgene on WT adult female offspring weight gain, body composition, and insulin sensitivity.** (A) WT female offspring weight gain. Offspring from each maternal group (*n* = 10 mothers per group) were weaned onto CD, and weighed every other week for 16 weeks. (B) Adult female offspring body composition (fat and lean mass) at 16 wks post-wean, as determined by Echo MRI. (C) Blood glucose curve in response to insulin tolerance test on fasted adult female offspring 16 wks post-weaning. All offspring measures are averaged per mother, with siblings treated as replicates. For Fat-1 mothers, only WT offspring were included in analyses. All data are expressed as mean ± SEM; *P<0.05.
(TIF)

Table S1 **Sequences of primers used for PCR-based genotyping and placental qPCR.**
(DOCX)
