# Supplementary figures and images for: Transgenic Increase in N-3/N-6 Fatty Acid Ratio Reduces Maternal Obesity-Associated Inflammation and Limits Adverse Developmental Programming in Mice
Source: PLoS One. 2013 Jun 25;8(6):e67791. doi: 10.1371/journal.pone.0067791 (PMC3692451; doi:10.1371/journal.pone.0067791)

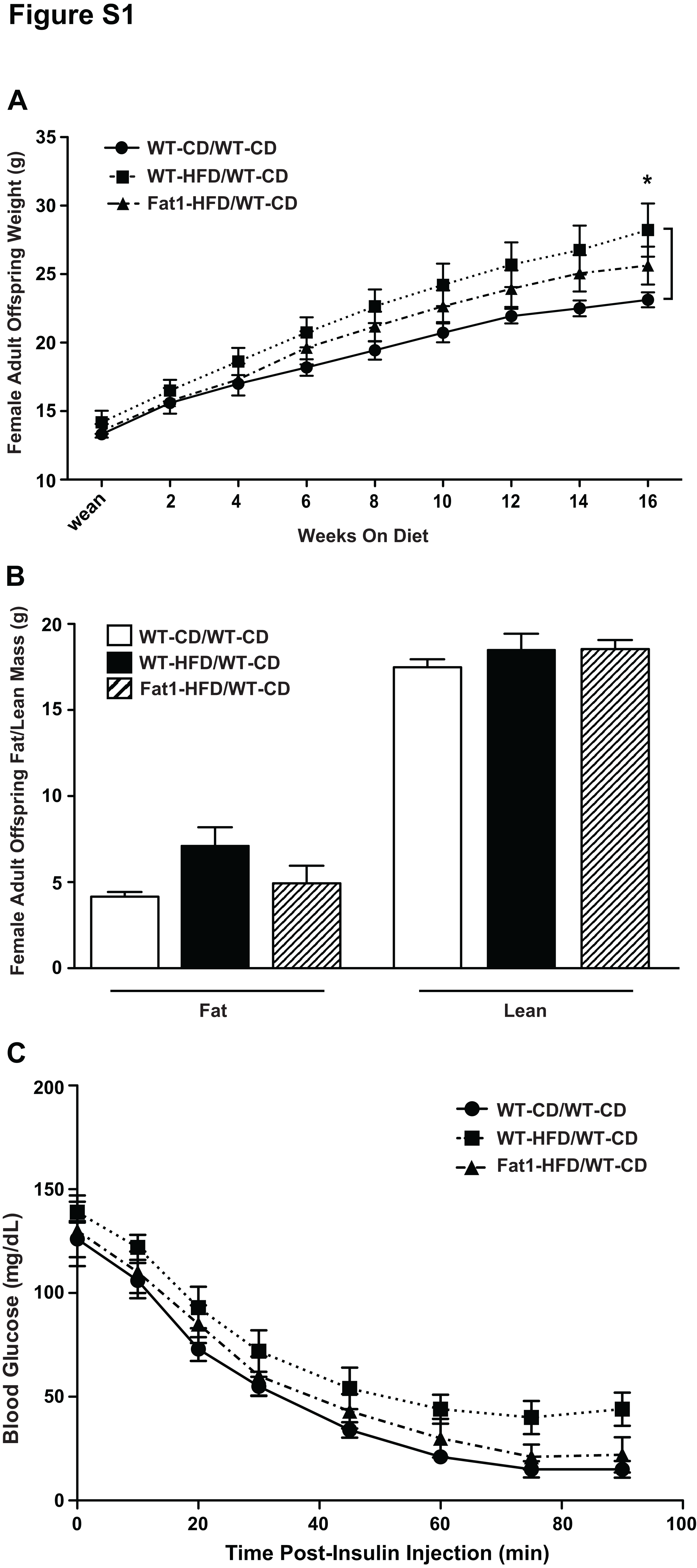

Supplement: Figure S1 — Effect of maternal HFD and Fat-1 transgene on WT adult female offspring weight gain, body composition, and insulin sensitivity. (A) WT female offspring weight gain. Offspring from each maternal group (n = 10 mothers per group) were weaned onto CD, and weighed every other week for 16 weeks. (B) Adult female offspring body composition (fat and lean mass) at 16 wks post-wean, as determined by Echo MRI. (C) Blood glucose curve in response to insulin tolerance test on fasted adult female offspring 16 wks post-weaning. All offspring measures are averaged per mother, with siblings treated as replicates. For Fat-1 mothers, only WT offspring were included in analyses. All data are expressed as mean ± SEM; *P<0.05. (TIF) [file pone.0067791.s001.tif]
