# Supplementary material for: Transgenic Increase in N-3/N-6 Fatty Acid Ratio Reduces Maternal Obesity-Associated Inflammation and Limits Adverse Developmental Programming in Mice
Source: PLoS One. 2013 Jun 25;8(6):e67791. doi: 10.1371/journal.pone.0067791 (PMC3692451; doi:10.1371/journal.pone.0067791)
Supplement: Table S1 — Sequences of primers used for PCR-based genotyping and placental qPCR. (DOCX) [file pone.0067791.s002.docx]

**Table S1. Sequences of primers used for PCR-based genotyping and placental qPCR.**

| **Gene** | **Forward Primer Sequence (5’-3’)** | **Reverse Primer Sequence (5’-3’)** |
| --- | --- | --- |
| Fat-1 | CTGCACCACGCCTTCACCAACC | ACACAGCAGCAGATTCCAGAGATT |
| SRY | TGGGACTGGTGACAATTGTC | GAGTACAGGTGTGCAGCTCT |
| TNFα | TCTCAGCCTCTTCTCATTCCTGCT | AGAACTGATGAGAGGGAGGCCATT |
| IL-6 | AGACAAAGCCAGAGTCCTTCAGAG | TTAGCCACTCCTTCTGTGACTCCA |
| IL-1β | GCCTTGGGCCTCAAAGGAAAGAAT | ATTGCTTGGGATCCACACTCTCCA |
| F4/80 | TCAAATGGATCCAGAAGGCTCCCA | TGCACTGCTTGGCATTGCTGTATC |
| iNOS | CTTGTGCTGTTCTCAGCCCAACAA | TCTGGAACATTCTGTGCTGTCCCA |
| Arg-1 | TGAGGAAAGCTGGTCTGCTGGAAA | TAGGGACATCAACAAAGGCCAGGT |
| LPL | ATGGATGGACGGTAACGGGAATGT | TGGATAATGTTGCTGGGCCCGATA |
| CD36 | TCATGCCAGTCGGAGACATGCTTA | AACTGTCTGTACACAGTGGTGCCT |
| FABP-4 | ATGAAATCACCGCAGACGACAGGA | TGTGGTCGACTTTCCATCCCACTT |
